# Draft Nuclear Genome Sequence of the Liquid Hydrocarbon–Accumulating Green Microalga *Botryococcus braunii* Race B (Showa)

**DOI:** 10.1128/genomeA.00215-17

**Published:** 2017-04-20

**Authors:** Daniel R. Browne, Jerry Jenkins, Jeremy Schmutz, Shengqiang Shu, Kerrie Barry, Jane Grimwood, Jennifer Chiniquy, Aditi Sharma, Thomas D. Niehaus, Taylor L. Weiss, Andrew T. Koppisch, David T. Fox, Suraj Dhungana, Shigeru Okada, Joe Chappell, Timothy P. Devarenne

**Affiliations:** a Department of Biochemistry and Biophysics, Texas A&M University, College Station, Texas, USA; b HudsonAlpha Institute for Biotechnology, Huntsville, Alabama, USA; c Joint Genome Institute, Walnut Creek, California, USA; d Plant Biology Program and Department of Pharmaceutical Sciences, University of Kentucky, Lexington, Kentucky, USA; e Department of Chemistry, Northern Arizona University, Flagstaff, Arizona, USA; f Chemistry Division, Los Alamos National Laboratory, Los Alamos, New Mexico, USA; g Laboratory of Aquatic Natural Products Chemistry, Graduate School of Agricultural and Life Sciences, the University of Tokyo, Bunkyo, Tokyo, Japan; h Japan Science and Technology Agency, Core Research for Evolutional Science and Technology (CREST), Chiyoda, Tokyo, Japan

## Abstract

*Botryococcus braunii* has long been known as a prodigious producer of liquid hydrocarbon oils that can be converted into combustion engine fuels. This draft genome for the B race of *B. braunii* will allow researchers to unravel important hydrocarbon biosynthetic pathways and identify possible regulatory networks controlling this unusual metabolism.

## GENOME ANNOUNCEMENT

The oil-producing traits of the colony-forming green microalga *Botryococcus braunii* have attracted study since the 19th century (1). In the late 20th century, morphologically similar strains were differentiated into three chemically distinct “races” (A, B, L) defined by the types of oils biosynthesized (alkadienes, botryococcenes, lycopadiene) in each race (2). *B. braunii* remnants have been identified in organic sediments dating from the Precambrian to the Permian period (3), indicating that *B. braunii* contributed material to petroleum formations (4). Like petroleum, *B. braunii* oil can be catalytically cracked, yielding fuel-range distillates (5, 6). Renewable fuel interests are currently driving *B. braunii* genomic research, and here we present an early release draft genome of the *B. braunii* race B (Showa) strain, which has an estimated size of 166.2 ± 2.2 Mb (7).

Genomic DNA was extracted and used to construct four Illumina libraries. First, a 2 × 250-bp paired-end library constructed from 800-bp fragments was sequenced to 700× coverage on an Illumina HiSeq 2500 platform. Second and third, 2 × 150-bp mate-pair libraries with 1.5-kb and 4-kb inserts were sequenced to 200× and 150× coverage on a HiSeq 2000. Fourth, a 2 × 300-bp mate-pair library with a 15-kb insert was sequenced to 3× coverage on an Illumina MiSeq. Two PacBio SMRTbell libraries were also constructed and sequenced to 200× coverage on the PacBio RS II platform.

The PacBio data were assembled with FALCON-Unzip, and the resulting sequences were polished using Quiver (8). To detect misassemblies, the 15-kb library was aligned to the sequences and clone coverage at each base computed. Nineteen misassemblies were detected and broken. The Illumina data were assembled with DISCOVAR. Sequences were identified in the DISCOVAR assembly that were not present in the FALCON assembly. The DISCOVAR assembly was masked using 24-mers from the FALCON assembly, and 487 unmasked sequences (1.396 Mb) were extracted. These sequences were combined with the broken FALCON assembly and scaffolded with the 15-kb library using SSPACE (9). Finally, the assembly was error-corrected using the Illumina data. Analysis revealed 523 scaffolds (19.8 Mb) that did not share a significant number of 24-mers with the rest of the assembly. These sequences were aligned to the NCBI NR database, identified as prokaryotic contamination, and removed from the assembly. Mitochondrial and chloroplast sequences (10) were removed prior to the assembly.

The final draft assembly consists of 184,385,342 bp in 2,752 scaffolds (*N*_50_ = 373 kb) with 49.6% G+C content and 1,148 gaps (4.611 Mb). There are 18,726 predicted genes with a mean of 5.7 exons per gene, a median exon length of 178 bp, and a median intron length of 578 bp. The 1,437 scaffolds with no genic content (the largest is 49,840 bp) account for 6,183,350 bp. This assembly provides a strong basis for functional and comparative analyses and will help elucidate the genetic basis of oil metabolism in *B. braunii*.

### Accession number(s).

This whole-genome shotgun project has been deposited at DDBJ/ENA/GenBank under the accession number MVGU00000000. The version described in this paper is the first version, MVGU01000000.
